# Regulation of cancer metastasis by microRNAs

**DOI:** 10.1186/s12929-015-0113-7

**Published:** 2015-01-23

**Authors:** Shih-Hsuan Chan, Lu-Hai Wang

**Affiliations:** Institute of Molecular and Genomic Medicine, National Health Research Institutes, 35 Keyan Road, Zhunan Town, Miaoli County 35053 Taiwan

**Keywords:** miRNAs, Breast cancer, Metastasis, Migration, Invasion, EMT

## Abstract

MicroRNAs (miRNAs) are a class of endogenous small non-coding RNAs that have been found highly conserved among species. MiRNAs are able to negatively regulate gene expression through base pairing of 3’ UTRs of their target genes. Therefore, miRNAs have been shown to play an important role in regulating various cellular activities. Over the past decade, substantial evidences have been obtained to show that miRNAs are aberrantly expressed in human malignancies and could act as “OncomiRs” or “Tumor suppressor miRs”. In recent years, increasing number of studies have demonstrated the involvement of miRNAs in cancer metastasis. Many studies have shown that microRNAs could directly target genes playing a central role in epithelia-mesenchymal-transition (EMT), a cellular transformation process that allows cancer cells to acquire motility and invasiveness. EMT is considered an essential step driving the early phase of cancer metastasis. This review will summarize the recent findings and characterization of miRNAs that are involved in the regulation of EMT, migration, invasion and metastasis of cancer cells. Lastly, we will discuss potential use of miRNAs as diagnostic and prognostic biomarkers as well as therapeutic targets for cancer.

## Introduction

MicroRNAs (miRNAs) are highly conserved small noncoding RNAs molecules naturally encoded in the genome of a variety of species. miRNAs function to affect RNA stability and translation to negatively regulate gene expression [1]. The miRNA maturation process requires several steps. Initially, miRNAs are transcribed as a form of long primary transcript (pri-miRNAs) from DNA by the RNA polymerase II (Pol II) or Pol III enzyme [2,3]. The long pri-miRNA transcript is processed by a nuclear RNase, Drosha, to generate pre-miRNAs with a stem-loop hairpin secondary structure [4]. Pre-miRNAs are then exported from nucleus to cytoplasm [5] where they are trimmed into mature miRNAs (22 ~ 25 nt) by the cytoplasmic RNase III, Dicer [6,7]. Mature miRNAs are then incorporated into the RNA-induced silencing complex (RISC) and exert their function by binding to the 3’ untranslated regions (3’UTRs) of their target genes. The binding could be either a partial complementarity, thereby blocking the translation, or in a perfect complementarity, leading to degradation of the target mRNA [8]. The imperfect match between miRNAs and their targets opens up the possibility for miRNAs to regulate multiple genes. The ability to modulate gene expression allows miRNAs to regulate various biological processes including differentiation, proliferation, angiogenesis and apoptosis [9]. Moreover, miRNAs have been shown to play a crucial role during caner development and progression in the past decade [10-16].

**Figure 1:**
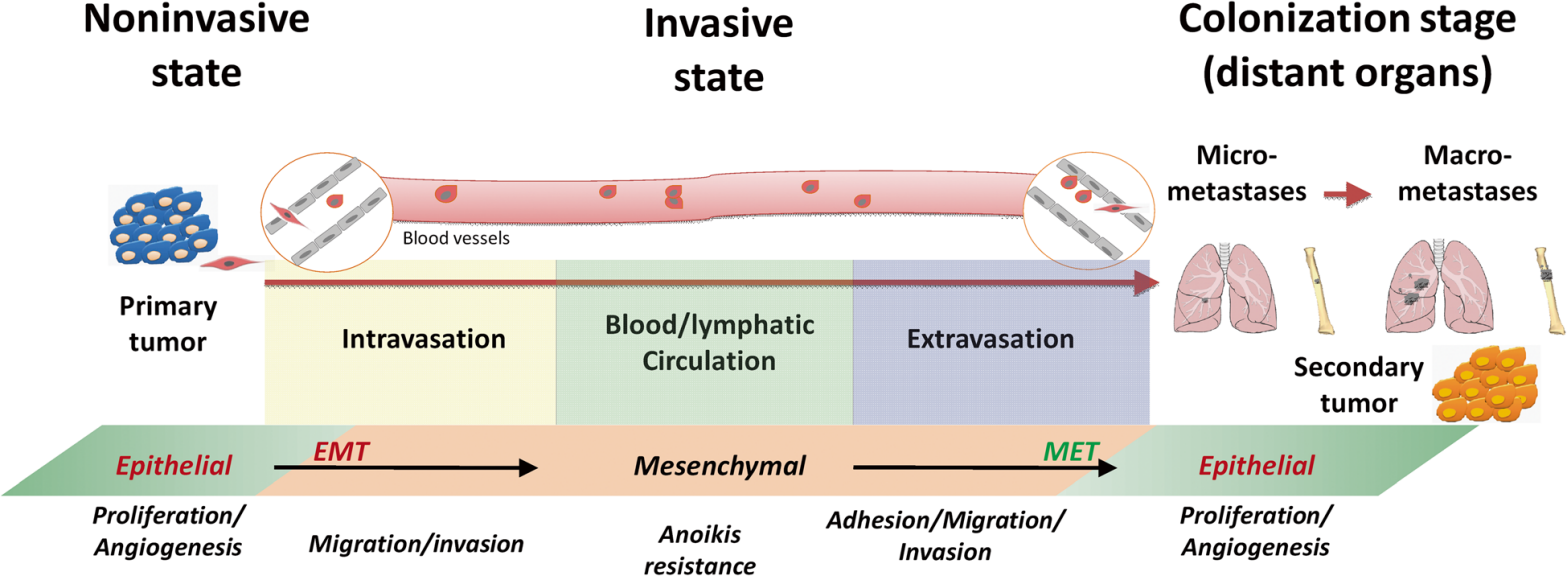
**The schematic of cancer metastasis: From primary site to disseminated organs.**

Cancer is among the diseases accounting for top mortality worldwide. An estimated 14.1 million new cases of cancer were diagnosed worldwide with 8.2 million deaths in 2012 [17]. More importantly, metastasis-related death accounts for 90% of cancer mortality [18-20]. Metastasis is an intricate multistep process that requires cancer cells to a) detach from the primary tumor and invade through basement membrane to nearby tissue; b) enter the blood vessels (intravasation); c) survive in the circulation (anchorage independent survival and growth); d) exit the circulatory system at metastatic sites (extravasation); and e) colonize and grow at the new environment and forming a metastatic tumor (colonization) [18,21] (see Figure 1).

Despite our increasing understanding of metastasis, there is still no effective ways or therapeutics to intervene metastatic processes. Gene signature and biomarkers including miRNAs that are reliable in predicting patients’ outcome in metastasis are just beginning to be unveiled.

For the role in cancer, Calin et. al first described that miRNAs were deregulated in human B cell chronic lymphocytic leukemia (CLL) by using a microarray containing hundreds of human precursor and mature miRNA oligonucleotide probes [22]. Since then miRNAs have been shown to be involved in the regulation of various cellular processes, and they are implicated in different diseases such as cardiac hypertrophy [23,24] , diabetes [25,26], Alzheimer’s disease [27,28], and hepatic viral infection [29,30]. MiRNAs including those in blood have been exploited as potential biomarkers for diagnosis and prognosis of diseases including cancers. Alteration of miRNAs expression in different types of cancers were subsequently reported [15,16,31]. Indeed, those miRNAs deregulated in cancers were later shown to act as oncogenes or tumor suppressors by suppressing their target genes [32,33]. In recent years, studies of miRNA functions and mechanisms have revealed their capacity to affect molecular pathways regulating epithelial-mesenchymal transition (EMT), which is a well-characterized cellular transition that is thought to be important at the initial step of cancer metastasis [34,35]. Clinical observations have shown altered expression of certain miRNAs correlating with poor prognosis of cancer [10,14,36-38]. Therefore, miRNAs have a potential to serve as cancer biomarkers [39-43].

In this review, we will focus on recent findings of miRNAs and their regulatory roles in cancer cell behaviors that affect metastasis and discuss the potential of miRNAs as biomarkers as well as miRNA-based therapeutics. The miRNAs known to be involved in distinct steps of metastasis including EMT, migration/invasion, anoikis survival, intravasation/extravasation and distant organ colonization will be discussed.

## Review

### Role of miRNA in the EMT/MET, migration/invasion and metastasis

#### microRNAs that control the EMT/MET processes

One of the most commonly accepted cellular transitioning processes that drives the early phase of cancer metastasis is the so-called epithelial-mesenchymal transition (EMT). EMT induces changes in the shape and motility of epithelial cells. Once transforming into mesenchymal phenotype, cancer cells lose their cell-cell contact and become mobile and invasive in order to spread into nearby tissues and subsequently distant organs [35,44]. Outgrowth at the site of distant dissemination requires metastatic cancer cells to undergo mesenchymal-epithelial transition (MET), a reverse process of EMT, where they regain epithelial properties [45-47].

**Figure 2:**
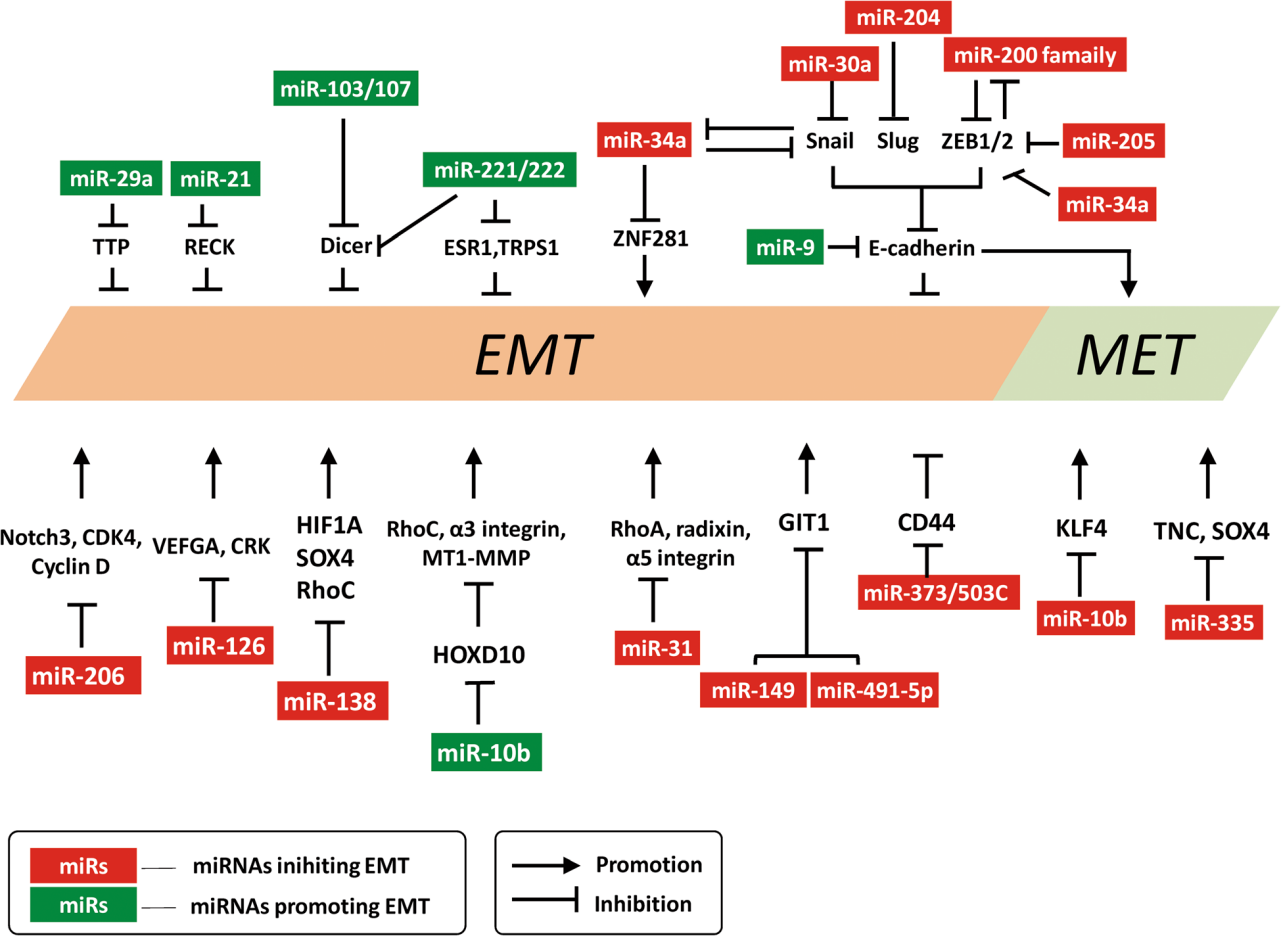
**MicroRNAs that are known to regulate EMT/MET processes and their validated targets.**

**Figure 3:**
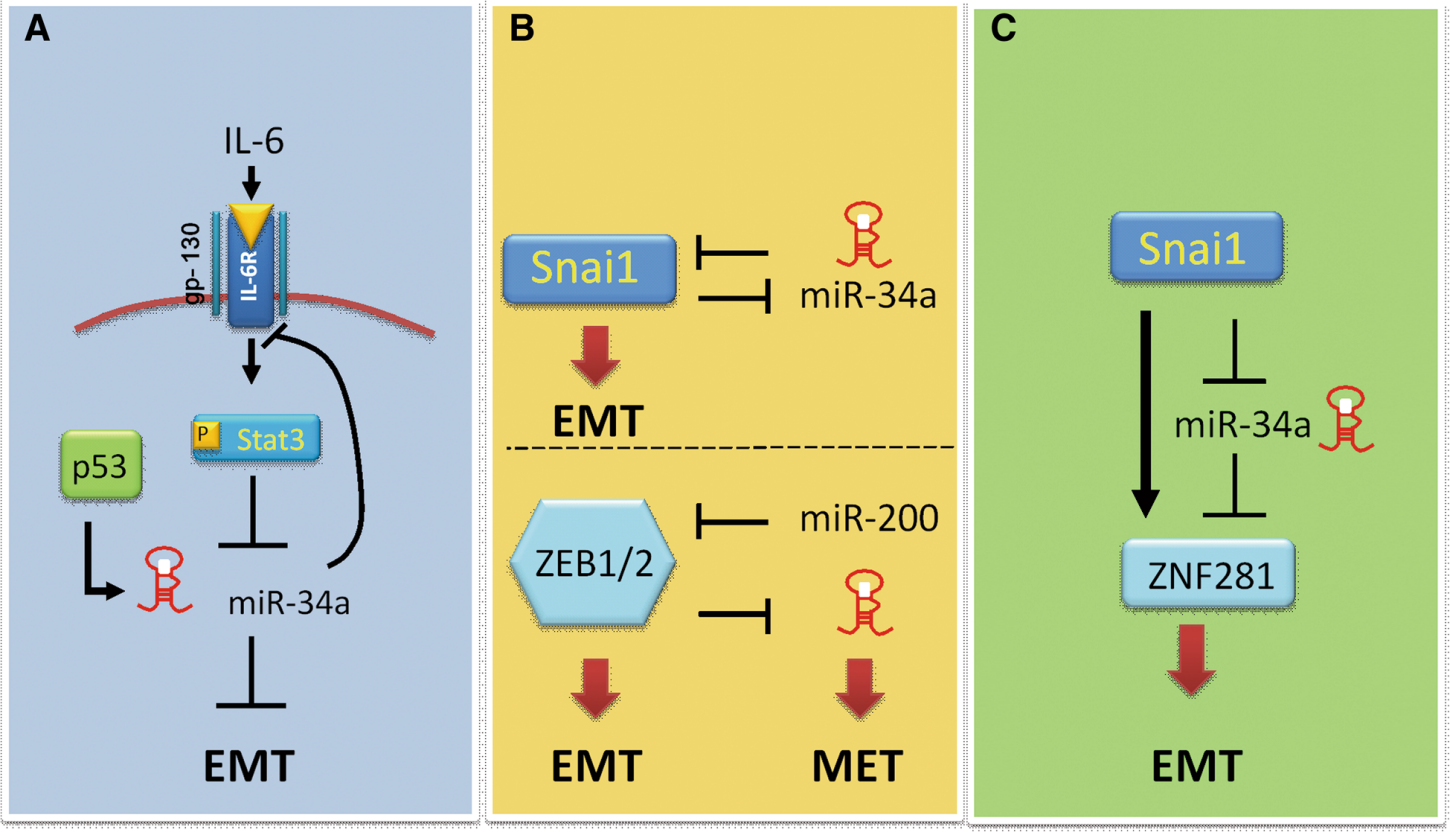
**Patterns of miRNA regulation circuits. (A)** Feedback loop. **(B)** Double feedback loop. **(C)** Feed forward loop.

Repression of E-cadherin expression in epithelial cancer cells is a hallmark for EMT. Several molecules are known to act as the repressor of E-cadherin expression including ZEB, Twist, Snail, Slug and TGF-β [48-54]. The miR-200 family (miR-200a/200b/200c/141/429) has been shown to inhibit cell migration and invasion through targeting ZEB in several cancer types including breast, bladder and ovarian cancers [55-58]. MiR-200 inhibition was reported to reduce E-cadherin level while promoting vimentin expression, thereby increasing cell motility [57]. Moreover, miR-200 and ZEB have been shown to form a reciprocal repression loop where ZEB repressed miR-200 expression while miR-200 targets ZEB [59] (see Figures 2 and 3). Thus, miR-200/ZEB plays a central role in the EMT/MET processes. Many reports further showed that ectopic expression of miR-200 family alone was enough to block TGF-β-induced EMT [56,57,60]. In non-small cell lung cancer cells and liver cancer cells, another miRNA, miR-30a, was found to inhibit EMT by targeting Snai1, thereby promoting E-cadherin expression [61,62]. In addition, miR-30a was also reported to suppress cell motility via targeting vimentin expression in gastric cancer cells [63]. In retinal pigment epithelium, miR-204/211 was shown to maintain epithelial barrier function by targeting TGF-β2 and Slug and thus is suppressor of EMT [64]. Siemens and colleagues showed that ectopic expression of miR-34a, a P53-regulated microRNA, could down-regulate Snail levels, therefore, leading to inhibition of EMT phenotypes including migration and invasion [65]. Furthermore, the same group demonstrated Snail bound to E-boxes in the promoter of miR-34a to suppress its expression. Therefore, miR-34a and Snail form a double negative feedback regulation loop to regulate EMT [65] (see Figures 2 and 3). In addition to EMT regulation, the well-known property of miR-34a is its tumor suppressor function via inducing cell cycle arrest and apoptosis in various cancer types by targeting several molecules crucial for sustaining tumor growth such as CDK4/6, MET, HDAC1, E2F3 and Bcl-2 [66-72] (Table 1).

**Table 1:** **miRNAs known to regulate EMT-related molecules**

**miRNAs**	**Role of EMT regulation**	**Target (s)**	**Cancer types**
miR-9	Positive	E-cadherin	Breast cancer [73], colorectal cancer [74]
miR-29a	Positive	TTP	Breast cancer [75]
miR-21	Positive	PTEN, RECK	Breast cancer [76], gastric cancer [77]
miR-103/107	Positive	Dicer	Breast cancer [78]
miR-221/222	Positive	Dicer, ESR1, TRPS1	Breast cancer [79]
miR-30a	Negative	Snail, Vimentin	Lung cancer [61], liver cancer [62], gastric cancer [63]
miR-34a	Negative	Snail, ZNF281, IL-6R	Colorectal cancer [65]
miR-200 family	Negative	ZEB1/2, ERRFI-1	Bladder cancer [58], breast cancer [56,57], ovarian cancer [55,57], lung cancer [57]
miR-204	Negative	Slug	Cholangiocarcinoma [64]
miR-205	Negative	ZEB1/2	Breast cancer [56]

miR-21, the first “oncomiR” to be identified, was shown to play a role in promoting EMT. Inhibition of miR-21 using antagomir in MDA-MB-231 invasive breast cancer cells was able to reverse EMT and cancer stem cell (CSC) phenotype by up-regulation of PTEN, leading to inactivation of AKT/ERK [76]. In gastric carcinoma, miR-21 was shown to directly target RECK (reversion-inducing-cysteine-rich protein with kazal motifs) expression to promote cell proliferation, migration and invasion [77]. miR-9, a MYC/MYCN-induced miRNA, has been demonstrated to directly target E-cadherin to promote breast cancer metastasis [73]. Clinical observations showed that miR-9 levels were increased significantly in primary breast tumors of patients with subsequent metastasis compared to those from metastasis-free patients [73]. miR-29a was also shown to induce EMT of Ras-transformed mouse mammary epithelial cells by targeting TTP (tristetraprolin) expression [75]. In a colon cancer study, miR-9 expression was activated by PROX1 (Prospero homeobox 1) and also leads to downregulation of E cadherin [74] (Table 1).

Dicer, an essential RNase III enzyme for microRNA biogenesis, was recently shown to be associated with EMT. Low level of Dicer was observed in breast cancer cells with a mesenchymal phenotype [80]. One study showed that miR-103/107 induced EMT by targeting Dicer expression, leading to the decrease of miR-200 [78]. Moreover, miR-103/miR-107 up-regulated ZEB levels in a miR-200-dependent manner [78]. The miR-221/222 cluster has also been shown to induce EMT in breast cancer cells by targeting Dicer, ESR1 (estrogen receptor 1) and TRPS1 (trichorhinophalangeal syndrome type I) [79] (Table 1). Most of those studies implicate the down-regulation of suppressor miRNAs to promote tumorigenesis.

Aside from EMT transition, a reverse process of EMT called MET (Mesenchymal-Epithelial-transition) has been shown to be important for the colonization of metastatic cells at distant organs. Recent reports have demonstrated the involvement of miRNA regulation in MET transition. Chen et al. showed that miR-103/107 directly targets MET inducer KLF4 and DAPK expression, leading to promoting metastasis [81]. miR-10b could also promote esophageal cancer metastasis by targeting KLF4 [82]. Those findings suggest that miRNAs also play an important role in MET transition of cancer cells.

#### miRNAs that regulate migration/invasion and metastasis

miR-10b was the first miRNA to be reported to play a promoting role in cancer metastasis. Ma and colleagues showed that overexpression of miR-10b in non-metastatic breast cancer cells induced invasion and distant metastasis by targeting HOXD10 mRNA, a transcriptional repressor that modulates several genes including RHOC, α3 integrin, uPAR and MTA-MMP (MMP14) [83]. In the same study, Twist, a well-known inducer of EMT, was identified to positively regulate miR-10b expression [83]. In glioblastoma cells, miR-10b-HOXD10 regulation axis and its downstream effectors, RHOC, uPAR and MTA-MMP also mediated invasiveness of cancer cells [84]. Another example of invasion/metastasis-promoting miRNAs is miR-373, which was initially considered as an oncomiR functioning to target LATS2, a tumor suppressor gene in testicular germ-cell tumors [85]. miR-373 and miR-503c stood out in a screening of metastasis-promoting miRNAs using a transwell migration assay [86]. These two miRNAs targeted the same downstream gene CD44 to stimulate MCF-7 cell migration/invasion *in vitro* and *in vivo* [86] (Table 2).

**Table 2:** **miRNAs involved in metastasis-related cell behaviors**

	**Role of metastasis regulation**	**Metastasis-relevant phenotypes**	**Target (s)**	**Cancer types**
miR-10b	Positive	migration, invasion, colonization	HOXD10, KLF4	Breast cancer [82,83]
miR-373/503c	Positive	migration, invasion	CD44	Breast cancer [86]
miR-31	Negative	migration, invasion	RhoA, radixin, α5 integrin	Breast cancer [87]
miR-126	Negative	migration, invasion, adhesion, angiogenesis	VEGFA, CRK	Lung cancer [88,89], gastric cancer [90]
miR-149	Negative	migration, invasion, adhesion	GIT1	Breast cancer [91]
miR-491-5p	Negative	migration, invasion, adhesion	GIT1	Oral cancer [92]
miR-138	Negative	migration, invasion	HIF1A, SOX4, RhoC	Ovarian cancer [93], kidney cancer [94], oral cancer [95]
miR-127	Negative	migration	BCL6	Breast cancer [96]
miR-206	Negative	migration	Notch3, CDK4, Cyclin D	Melonoma [97], Cervial cancer [98]
miR-335	Negative	migration, invasion, colonization	SOX4, TNC	Breast cancer [99]

Aside from a few pro-metastatic miRNAs, growing evidence showed that a greater number of miRNAs act as suppressors of migration/invasion and metastasis. miR-31, a pleiotropically acting miRNA, inhibits different stages of metastasis including local invasion, anoikis resistance, extravasation and metastatic colonization. Three pro-metastatic genes, RHOA, radixin and α5 integrin were found to be directly targeted by miR-31 in breast cancer cells [87]. Tavazoie and colleagues showed that restoring the expression of miR-335, miR-126 or miR-206 through retroviral transduction significantly reduced the ability of CN34-LM1 and CN34-BoM1 cells to metastasize to lung and bone, respectively [99]. Among those miRNAs, the low expression levels of miR-335 were associated with very poor overall metastasis-free survival in comparison with patients whose tumors expressed a high level of this miRNA [99] (Table 2). It was demonstrated that miR-335 suppressed migration/invasion through targeting the progenitor cell transcriptional factor SOX4 and extracellular matrix component tenascin C (TNC) [99]. In addition, another well-characterized miRNA that possesses a suppressor function is miR-138. A number of research groups have demonstrated that miR-138 is not only involved in tumorigenesis [100,101] but also regulating metastasis-related events such as migration and invasion by targeting several downstream genes including RhoC, HIF1α and SOX4 in different context of cancer cells [93-95]. Other studies demonstrated that miR-206 induced apoptosis and inhibited cell migration through modulating expression of Notch3, CDK4 and Cyclin D [97,98]. MiR-126, on the other hand, affects cancer cell migration, adhesion and angiogenesis through modulating proangiogenic factor VEGFA [88] and an adaptor protein Crk [89,90] (Table 2).

Two recent studies showed that GIT1 (G protein-coupled receptor kinase interacting ArfGAP 1), an important scaffold protein for focal adhesion complexes, plays an important role in cancer cell migration/invasion and metastasis [91,92]. GIT1 could be directly targeted by miR-149 and miR-491-5p in the different context of cancer cells [91,92]. In breast cancer, Chan and colleagues showed that GIT1 was a direct target of miR-149 and was down-regulated by this miR, leading to instability of α5β1 integrins and paxillin. As a result, miR-149 suppresses the ability of migration/invasion and lung metastasis of the highly metastatic breast cancer line, MDA-MB-231-IV2. Clinical analysis showed that miR-149 was decreased while GIT1 level was increased in lymph node metastases compared to the matched primary breast tumors [91]. In OSCC (oral squamous cell carcinoma), Huang et al. showed that miR-491-5p inhibited migration/invasion and metastasis of oral cancer cells and this was also through targeting GIT1 expression. Moreover, low level of miR-491-5p and high level of GIT1 were correlated with lymph node metastasis and overall survival of OSCC patients [92]. Thus, miR-149 and miR-491-5p are potent metastasis suppressors in breast and oral cancer respectively. The finding of distinct cancer types with different miRs targeting the same substrate GIT1 to inhibit metastasis implies the important role of this molecule in cancer cell migration/invasion and metastasis (Table 2).

The let-7 family, first discovered in *Caenorhabditis elegans*, was found down-regulated in a variety of human malignancies [102-104]. Recent reports provided a link between let-7 and cancer metastasis. In a lung cancer study, let-7 was initially found to reduce oncogenic proteins RAS and HMGA2 [105,106]. Later, overexpression of let-7 was shown to suppress mammosphere-forming ability *in vitro* and metastatic potential *in vivo*. Reducing let-7 levels in breast cancer initiating cells could promote tumoregencity and metastatic ability in a NOD/SCID mouse xenograft model [107]. High levels of RAS and HMGA2 were found in breast cancer initiating cells and were inversely correlated with let-7 expression [107]. Thus, let-7 plays a central role in breast cancer stemness and metastasis. In addition, let-7 has been shown to be inhibited by miR-107. MiR-107 was shown to directly interact with mature let-7 to inhibit its function, leading to promoting tumor progression and metastasis [108] (Table 2).

**Figure 4:**
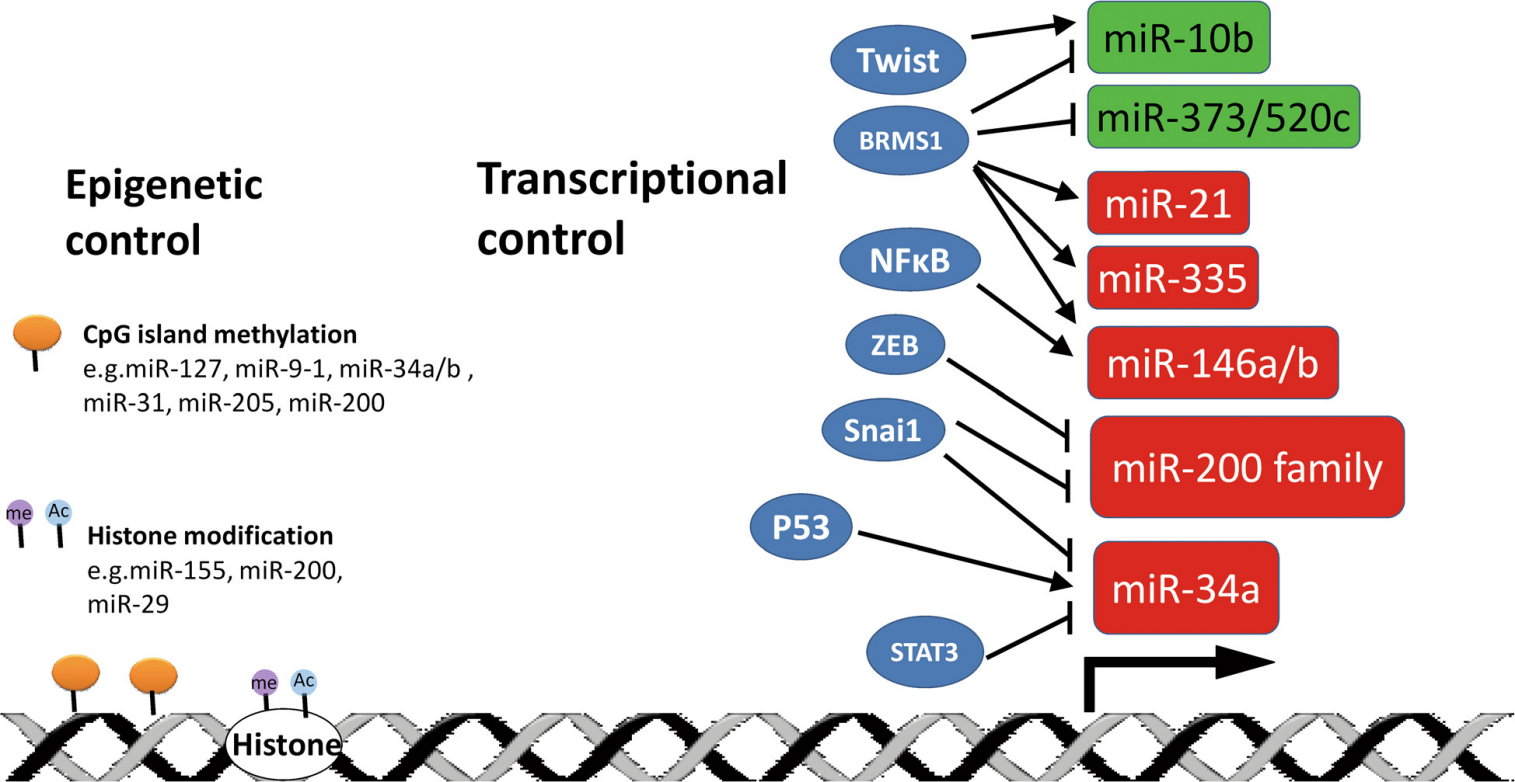
**Upstream transcriptional regulation of microRNAs.**

Besides the miRNA-regulated gene networks during metastatic processes, the transcriptional control of metastasis-relevant miRNAs has been recently unraveled. It has been shown that breast cancer metastasis suppressor 1(BRMS1) plays an important role in the transcription of several metastasis-relevant miRNAs. BRMS1 was shown to act as a repressor for metastasis-promoting miRs such as miR-10b, miR-373 and miR-520c and an activator for metastasis-suppressing miRs miR-146a/b, miR-335 and miR-21 [109] (Figure 4). Moreover, BRMS1 also could decrease Twist, which is upstream of miR-10b [83]. Several studies have indicated that miRNAs frequently form feedback loops, since they could be regulated by transcription factors, which they directly or indirectly target [110,111]. A good example of miRNA-regulated feedback loop in cancer metastasis is NF-кB/miR-146 signaling [112]. Previous studies have shown that miR-146 induction depends on NF-кB activation [113]. A subsequent investigation demonstrated that miR-146a/b suppressed breast cancer metastasis via reducing the activity of NF-кB by directly targeting IRAK1 and TRAF6, both of which are known to positively regulate NF-кB activity [114]. Those findings suggest that NF-кB and miR-146 form a negative regulatory loop. Recently, Rokavec and coworkers uncovered a feedback loop formed by IL-6/STAT3-mediated represssion of miR-34a and upregulation of IL-6R (IL-6 receptor), which promotes EMT-mediated colorectal cancer invasion and metastasis [115] (see Figure 3). As described above, miR-200/ZEB and miR-34a/Snail were found to form a reciprocal suppression loop where miRNA targets could repress miRNAs themselves [59,67] (see Figures 2 and 3). Further complicating the transcriptional regulatory circuit of miRNAs, there are evidences showing that there exists a coherent“feed-forward”loop (FFP) in the Snail-miR-34a-ZNF281 regulation axis where Snail could both regulate miR-34a and ZNF281 expression but in a negative and positive manner respectively [116] (see Figure 3). In this feed-forward loop, Snail was found to suppress miR-34a while promoting ZNF281 expression, leading to the induction of EMT.

Aside from the transcriptional control, altered expressions of miRNAs could be a result of epigenetic changes. An extensive analysis of genomic sequences of miRNA genes has revealed that approximately half of them are associated with CpG islands [117]. Indeed, many studies have indicated that methylation status could be responsible for the deregulated expression of miRNAs in cancers. Saito and colleagues showed that silencing of miR-127 in several cancers was due to promoter hypermethylation [118] and treatment of a bladder cancer cell line with DNMT inhibitor 5-Aza-2’-deoxycytidine could strongly up-regulate the miR-127 level and down-regulate BCL-6 expression, which was shown to be directly targeted miR-127 [118]. It has been also shown that miR-9-1 and miR-34a/b are hypermethylated in breast and colon cancer respectively [119,120], In addition, histone acetylation was also reported to play a role in regulating miRNA expressions. Several reports have shown that HDAC inhibitors altered miRNA expression in human cancer cell lines including breast [121] and colon cancers [122,123].

### miRNAs as diagnostic and prognostic biomarkers for cancer growth and metastasis.

Cancer patients who are diagnosed at an early stage usually have a better prognosis and overall survival rate [124]. In this regard, the need to develop effective early biomarkers as well as those for predicting treatment outcome cannot be overemphasized.

Given that miRNAs are relatively stable due to their small size, it raises the possibility that analysis of miRNA expression may be a useful tool to define cancer states. As described earlier, miRNA expression has been found deregulated in a variety of human malignancies [15,16]. Lu et al. was the first to demonstrate that the expression pattern of miRNAs could further classify cancer types [15]. Moreover, miRNA profiles not only could be used to distinguish normal from cancerous tissues, but could also distinguish different subtypes of breast cancer. Blenkiron et al. have shown that high level of miR-200 family correlates with the luminal type whereas miR-205 and miR-145 are greatly reduced in the basal-like triple negative breast cancer (ER^−^/PR^−^/HER^−^) [125]. Several groups have reported miRNA expression signature in predicting cancer outcome. The first evidence came from the study of Calin et al. who reported the unique miRNA signature associated with progression and prognosis of chronic lymphocytic leukemia (CLL) [22]. In lung cancer, downregulation of miR-155 and let-7a-2 was reported to predict poor prognosis [126]. Recently, more and more miRNAs as prognostic biomarkers have been reported in various cancer types. A 7-miRNA classifiers can be used to predict patients’ overall and relapse-free survival in gastric cancer [38]. Similarly, a microRNA signature was reported to be able to predict survival and relapse in lung cancer patients [127]. Low miR-191 and high miR-193a levels were shown to be associated with shorter survival in melanoma patients [36]. The miR-21, a well-studied oncomiR, also has been shown to serve as an indicator of poor prognosis in various cancer types, including breast [128,129], liver [130], lung [131], and colorectal cancer [132]. A hypoxia-induced miRNA, was identified as a prognostic marker for breast cancer patients. High miR-210 expression was shown to have an inverse correlation with disease-free and overall survival for breast cancer patients [133]. Several studies reported that certain miRNAs could be used as biomarkers to predict cancer metastasis. Primary tumors with low levels of miR-335 and miR-126 have a higher probability of developing metastasis at secondary sites in breast cancer patients [99]. In OSCC, tumors with low levels of miR-491-5p have a higher tendency to form lymph node metastasis [92]. Similarly, miR-149, a potent metastasis suppressor, was found to be down-regulated in metastatic tumors of breast cancer patients [91].

Recently, a growing body of evidence has indicated that circulating miRNAs could serve as biomarkers for cancer prognosis [134-136]. Indeed, circulating miRNAs have been extracted and detected from a variety of samples including blood (plasma or serum) [43,134], urine [137], saliva [138,139] and sputum [140,141] . They have great potentials to serve as novel biomarkers for early diagnosis and prognosis of cancer. For example, serum levels of miR-92a and miR-29a are significantly increased in patients with colorectal cancer [142]. The levels of miR-141 in the serum can distinguish healthy people from prostate cancer patients [134]. Those studies open up new strategies for cancer detection and follow-up disease management.

Besides the value of miRNAs as biomarkers for predicting survival and disease progression, recent studies have revealed another great potential of miRNAs as parameters for predicting the responses of cancer patients to specific therapies. For instance HCC (hepatocarcinoma) patient with a low miR-26 level responded well to interferon-α treatment resulting in improved survival [143]. On the other hand, increased miR-21 could predict poor response to adjuvant chemotherapy in colorectal [132,144] and lung cancer patients [39].

Therefore, different expression levels of certain miRNAs could be used to discriminate patients who could benefit most from particular therapies.

### miRNAs as therapeutic targets and tools

Gain- and loss-of-function studies of miRNAs have provided insights towards the possible use of miRNAs in therapeutic interventions. The fact that a single miRNA has multiple target genes requires careful considerations when using miRNAs as therapeutics. The positive aspect is its capability to targeting multiple related pathways. The downside is the off targeting and concern of specificity.

There are at least two possible approaches to manipulate miRNA expression in cancer cells. Notably, 1) miRNA-based therapy: Introduction of antisense miRNAs (Anti-miRs) to block the function of oncogenic miRNAs/metastasis-promoting miRNAs or re-introduction of synthetic miRNAs (miR mimics) to mimic tumor suppressor or metastasis suppressor miRNAs that are reduced or lost in cancer cells. 2) Induction of miRNAs expression: This strategy involves the use of drug to control miRNA expression by modulating its transcription or processing.

Stability and effective delivery to target sites remain the major challenge for miRNA-based therapy and their optimization is the key for the success of this kind of approach. Given that therapeutic miRNAs would be systemically delivered into the blood stream, some modifications need to be made to prevent them from being filtered by kidney (molecules less than 52 kDa would be filtered and excreted in urine. An estimated size of unmodified dsRNAs is 7 ~ 20 kDa) and they are removed or damaged by nucleases and phagocytic immune cells such as macrophages [14]. Several chemical modifications have been used *in vivo* to date. Two major chemical modifications, 2’-O-methyl-group (OMe)-modified oligonucleotides [145,146] and locked nucleic acid (LNA)-modified oligonucleotides [147,148], have been widely applied to enhance stability of oligonucleotides. More comprehensive types of modification have been previously reviewed [1,149]. In addition, a modification of oligonucleotides at 3’ end using cholesterols has been demonstrated to greatly improve their cellular uptake [150,151].

Calin et al. provided the first indication of the feasibility of miRNA-based cancer therapeutics. Re-introduction of miR-15a/16-1 caused apoptosis in leukemic MEG01 cells and suppressed tumor growth in a xenograft model [152]. In breast cancer, Ma et al. demonstrated the potential therapeutic application of silencing a metastasis-promoting miR, miR-10b, in a mouse model. They reported that systemic treatment of tumor-bearing mice with miR-10b antagomir, a 2’-O-methyl-group (OMe)-modified, cholesterol-conjugated antisense miR could suppress breast cancer metastasis [153]. Tazawa and colleagues reported that systemic delivery of miR-34a mixed with atelocollagen inhibited human colon cancer progression [154]. Another group developed a LPH (liposome-polycation-hyaluronic acid) nanoparticle formulation modified with tumor-targeting single chain antibody fragment (scFv) for systemic delivery of miR-34a in a murine B6F10 lung metastasis model and showed reduced tumor load in the lung [155]. To date, miR-34a mimics MRX34 is the first miRNA mimicry to be advanced to human clinical trial (http://clinicaltrials.gov/ct2/show/NCT01829971). Moreover, researchers have demonstrated another possible use of miRNAs as adjuvant agents [156,157]. Overexpression of miR-205 in SKBR3 breast cancer cells could increase their responsiveness to tyrosine kinase inhibitors Gefitinib and Laptatinib by suppressing HER3 [158]. Another way to increase the endogenous expression of miRNA of interest is by the use of adenoassociated viruses (AAV). One major advantage of using AAV as a viral vector for delivery is its availability of a number of different AAV serotypes, which allow for the potential tissue-specificity due to the property of each serotype [159]. Kota et al. reported that AAV-mediated delivery of miR-26a alleviated tumorigenesis in a mouse liver cancer model [160].

Taken together, those reports suggest that manipulating miRNA expression could be an approach for cancer treatment and miRNA-based therapeutics in combination with other cancer drugs could also be considered for improved new regimens.

## Conclusion

With over 10 years of extensive studies of miRNAs including expression profiling, action mechanism, functional characterization and clinical implication, cancer biologists have unraveled the fundamental role of miRNAs in cancer progression and metastasis. In the next decade, the challenge of miRNAs research would probably be how the bench discoveries so far could be translated into clinical application. In this respect, designs and modifications to increase stability of miRNA mimics and anti-miRs should be optimized, and methods to improve the efficiency and specificity of *in vivo* delivery to target organs and types of cells need to be developed. Despite the potential complication of miRNA-based therapies, in which a miRNA may have unexpected off targets that lead to an unpredicted result, the current discoveries of miRNAs and anti-miRs as a new class of drug targets are encouraging and provide a promising therapeutic strategy for interventions of cancer progression and metastasis.
